# Cardiac device motion tracking from kilovoltage projections during stereotactic arrhythmia radioablation

**DOI:** 10.1016/j.phro.2026.100922

**Published:** 2026-02-10

**Authors:** Wenjuan Xiong, Doan Trang Nguyen, Daryl Wilding‐McBride, Paul J. Keall, Chandrima Sengupta, Melanie Grehn, Hofmaier Jan, Stefanie Corradini, Mirko Fischer, Merten Roland, Oliver Blanck, Judit Boda‐Heggemann, Lena Kaestner, Ricky O’Brien

**Affiliations:** a School of Health and Biomedical Sciences, RMIT University, Victoria, Australia; b Image X Institute, The University of Sydney, New South Wales, Australia; c SeeTreat Medical, New South Wales, Australia; d Department of Radiation Oncology, University Medical Center Schleswig‐Holstein, Kiel, Germany; e Department of Radiation Oncology, University Hospital of the Ludwig‐Maximilians‐University, Munich, Germany; f Department of Radiation Oncology, Hannover Medical School, Hannover, Germany; g Department of Radiation Oncology, University Medical Center Mannheim, Medical Faculty Mannheim, University of Heidelberg, Mannheim, Germany; h DKFZ Hector Cancer Institute at the University Medical Center, Mannheim, Germany

**Keywords:** Cardiac motion, Respiratory motion, Ventricular tachycardia, Kilovoltage images, Stereotactic arrhythmia radioablation

## Abstract

**Background and purpose:**

Cardiac and respiratory motion present a challenge to accurate dose delivery during stereotactic arrhythmia radioablation (STAR). This study investigated a motion tracking method using kilovoltage (kV) images on a gantry‐mounted linear accelerator during STAR.

**Materials and methods:**

An implantable cardioverter‐defibrillator (ICD) lead tip was used for motion tracking. Planning computed tomography and cone beam computed tomography scans were analysed retrospectively on ten patients treated with STAR in RAdiosurgery for VENtricular TAchycardia (RAVENTA) (four patients underwent deep inspiratory breath‐hold, two with free breathing, three under abdominal compression, and one using high frequency ventilation). A template‐matching approach was developed to segment ICD lead tips in kV projections, with successful segmentation defined as a segmented position within 2 mm of the two‐dimensional (2D) ground truth derived from manual annotations. A probability distribution function method was used to convert 2D coordinates to three‐dimensional (3D) trajectories. 3D trajectories from the 2D ground truth were used to measure ICD lead tip displacements from its mean position. Differences between 3D trajectories from 2D ground truth and segmentations were analysed.

**Results:**

The median (range) 2D segmentation success rate was 85% (71–96%). Median (range) motion displacements were 1.4 (0.1–7.4) mm (left‐right), 1.5 (0.1–10.4) mm (superior‐inferior), and 0.8 (0.0–6.3) mm (anterior‐posterior). The median (range) proportion of 3D estimations with errors below 2 mm in all axes was 78% (43–96%).

**Conclusions:**

ICD lead tip motion showed patient‐specific variability. This study presented a potential 3D motion tracking method for ICD lead tips during STAR.

## Highlights

• Cardiac and respiratory motion tracking method for cardiac radioablation.
• Implantable cardioverter-defibrillator lead tip segmentation with 85% accuracy.
• Three-dimensional motion estimation with errors < 2 mm in 78% of cases.
• Left–right, superior–inferior, and anterior–posterior motions: 7.4, 10.4, and 6.3 mm.

## Graphical abstract

**Figure:**
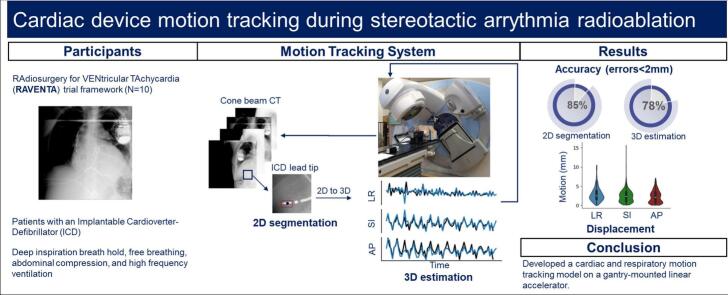

## 1 Introduction

Ventricular tachycardia (VT) is a common arrhythmia affecting millions of people worldwide [1]. Patients with VT are at substantial risk of sudden cardiac death [2]. The main treatment for refractory VT is catheter ablation; however, this invasive procedure may not be suitable for all patients due to potentially unfavorable outcomes and significant risks [3].

Stereotactic arrhythmia radioablation (STAR) has emerged as a promising treatment for refractory monomorphic VT, demonstrating encouraging clinical results [4], [5], [6]. The rapid and asymmetrical nature of heartbeats, combined with respiratory motion, makes target motion more complex than conventional targets in radiotherapy [7], [8]. Cardiac and respiratory motion during radiation treatment, which remains incompletely understood, could introduce substantial inaccuracies and safety risks [9]. To mitigate challenges, motion management techniques, including breath holds, internal target volumes, and gated deliveries, have been proposed; however, their effectiveness requires further evaluation [8].

Studies have investigated cardiac and respiratory motion for patients with VT using electrocardiogram (ECG)‐gated computed tomography (CT) [7], [10], [11], [12], [13], respiratory‐gated CT [11], [12], [13], [14], magnetic resonance imaging [15], [16], and synchrony imaging in the CyberKnife [17]. These studies have demonstrated that motion varies across patients [7], [12]. Real‐time motion data could provide valuable insights into motion variations during STAR. Challenges include difficulty of visualising heart structures without a contrast agent and significant deformation and movement of the heart due to heartbeats and respiration. Therefore, it is crucial to develop a model for tracking cardiac and respiratory motion during STAR.

As the treatment target is generally not visible on X‐ray imaging, surrogate tracking provides a practical alternative. Implanted markers as surrogates have been used for lung tumor tracking during stereotactic body radiotherapy trials, and the geometric uncertainty has been quantified [18]. STAR patients often have at least one implantable cardioverter‐defibrillator (ICD) lead in the right ventricle (RV) from prior treatment, which may be anchored near the interventricular septum and adjacent to left ventricular targets [19]. Compared to the diaphragm, the ICD lead tip, residing within the heart, offers a more reliable surrogate for motion tracking [20]. Furthermore, studies demonstrated a correlation between ICD lead and treatment target motion, with a majority of patients showing this relationship under breathing [12], [21]. These findings supported the feasibility of using ICD lead tip for motion tracking during STAR.

This study focuses on constructing an ICD lead tip motion tracking model using kilovoltage (kV) images on a gantry‐mounted linear accelerator. Cone beam CT (CBCT) projections from STAR patients across different motion management strategies were analysed. Key contributions include the development of a two‐dimensional (2D) segmentation method for ICD lead tips and the application of 2D to three‐dimensional (3D) conversion techniques for motion tracking.

## 2 Materials and methods

### 2.1 Patient and imaging data

Anonymized planning CT and pre‐treatment CBCT scans of patients treated within the German multi‐center multi‐platform RAdiosurgery for VENtricular TAchycardia (RAVENTA) trial framework (NCT03867747) [22], [23] were retrospectively analyzed. Technical parameters of the CT dataset and CBCT system were listed in previous work [24]. Ten patients had at least one ICD lead tip in the RV; for three with multiple RV leads, the tip nearest the clinical target volume was selected. A total of fifteen CBCT scans with a median (range) of 368 (184–714) projections per scan were analysed. Four patients underwent deep inspiratory breath‐hold (DIBH) scanning (nine scans), two were scanned during free breathing (FB) (two scans), three under abdominal compression (AC) (three scans), and one intubated patient in the intensive care unit using high‐frequency ventilation (HFV) (one scan).

### 2.2 Two-dimensional segmentation

**Fig. 1:**
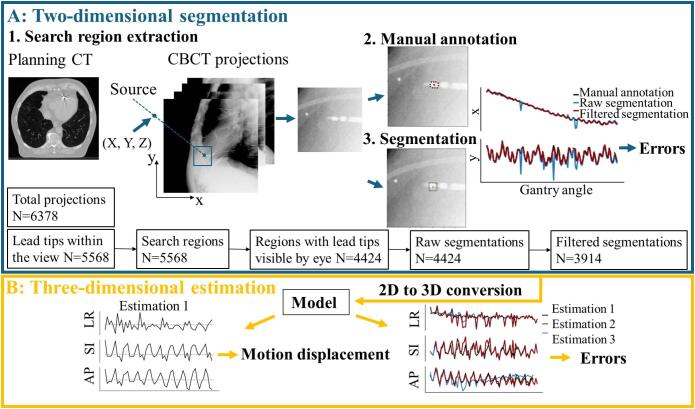
Study overview. (A) 2D segmentation. Step 1: A search region (70 × 70 pixels) was extracted from each projection; Step 2: The centroid of manual annotations (red dot) corresponds to the 2D ground truth; Step 3: The centroid of the box (red dot) from a template matching method corresponds to the segmented position. (B) 3D estimation. Model represents the 3D estimation model. Black trajectories (Estimation 1) represent 3D motion estimated from the 2D ground truth, used to measure motion displacement. Grey dashed lines represent the mean position, calculated by averaging the positional values over all time points. Red (Estimation 2) and blue trajectories (Estimation 3) represent motion estimated from filtered and raw segmentations. Abbreviation: 2D = two‐dimensional; 3D = three‐dimensional; CT = computed tomography; CBCT = cone beam computed tomography; LR = left‐right; SI = superior‐inferior; AP = anterior‐posterior. (For interpretation of the references to colour in this figure legend, the reader is referred to the web version of this article.)

The study involved 2D segmentation of ICD lead tips, followed by 3D motion estimation (Fig. 1). Two‐dimensional segmentation consisted of three steps. In the first step, a 70 × 70 pixels search region was defined for each projection. The 3D lead tip coordinate relative to the isocenter from planning CT scans, was projected onto the 2D imager plane using the geometric parameters during the CBCT scan. A search region centered at the projected position was defined for each angle. In the second step, search regions were manually annotated using Visual Geometry Group Image Annotator (v2.0.12) [25]. Two human annotators visually inspected and delineated the lead tip region with a polygon and used its centroid as the 2D ground truth. Search regions in which lead tips were not visible were excluded. A median (range) of 303 (122–447) search regions per scan containing annotated lead tips were generated, accounting for a median (range) of 70% (49–100%) of projections. In the third step, template matching segmentation was conducted within each search region. A template was constructed by projecting a cylinder (geometry determined by the configuration of each lead tip, Supplementary Table S1) onto an imager plane from each angle. To account for motion due to heartbeats and breathing, the template was rotated eight times for DIBH and twenty times for FB, AC, and HFV scans at 5° intervals, generating a set of templates. Normalised cross‐correlation (NCC) was computed by sliding templates across the search region. The position, orientation, and size with the highest NCC coefficient was identified as the segmented position. The distances between the 2D ground truth (the centroid defined by human annotators) and the centroid of the segmented area (the position identified through NCC) were measured. Segmentation was deemed successful if the distance was less than 2 mm (slice thickness in CT scans) in both x‐ and y‐directions. The fraction of successful segmentations was calculated.

### 2.3 Three-dimensional estimation

Two-dimensional to three-dimensional conversion was based on a Gaussian probability distribution function (PDF) determined via maximum likelihood [26]. 3D trajectories estimated from the 2D ground truth (Estimation 1) were used to measure motion displacement. For each CBCT scan, a mean position was calculated by averaging positional values over all time points. The absolute difference between each positional value and the mean was defined as motion displacement. Motion displacements were summarized by the 5th–95th percentile range, excluding the top and bottom 5% of values.

To compute 3D estimation errors, the trajectory estimated from the 2D ground truth (Estimation 1) was used as reference, and the trajectory estimated from segmentations (Estimation 2) was used for assessment. The difference between the two trajectories was evaluated. To prevent large 2D segmentation errors into the 3D conversion, a filtering step was applied: the mean superior‐inferior (SI) pixel value across segmentations was calculated, and any segmentation deviating by more than 25 pixels/20 mm from this mean was rejected. To support real‐time feasibility, the mean values were updated iteratively as new segmentations were obtained. This threshold was chosen based on the largest deviations observed in the 2D ground truth. The remaining filtered segmentations were used for 2D‐to‐3D conversion, and the resulting estimation errors were calculated. For comparison, the estimation errors from the non-filtered segmentations (Estimation 3) were also reported. The fraction of 3D estimations with errors less than 2 mm in all axes was evaluated. Statistical significance between errors from non‐filtered and filtered segmentations was assessed using the Wilcoxon signed‐rank test.

## 3 Results

The median (range) success rate for 2D segmentations was 80% (58–94%) (Table 1). After applying the filtering step, the median (range) success rate increased to 85% (71–96%) with a median filtering rejection rate of 7% (Wilcoxon signed‐rank test, p < 0.01). Large errors occurred in P1 (−3.7 mm and 5.1 mm), P6 (7.5 mm), and P10 (3.7 mm).

**Table 1:** Two‐dimensional segmentation results across motion management groups. Accuracy: the fraction of segmentations with errors < 2 mm in both x‐ and y‐directions.

Segmentation accuracy (%)															
	Deep inspiratory breath‐hold									Free breathing		Abdominal compression			High‐frequency ventilation
Patient	P1			P2		P3		P4		P5	P6	P7	P8	P9	P10
Scan	1	2	3	1	2	1	2	1	2	1	1	1	1	1	1
Non-filtered	84	82	80	87	87	94	80	77	72	90	58	78	76	73	71
Filtered	86	85	82	92	90	96	87	78	73	91	71	84	81	88	84

Segmentation errors (5th–95th percentile)								
	Deep inspiratory breath‐hold		Free breathing		Abdominal compression		High‐frequency ventilation	
	x‐direction y‐direction	x‐direction y‐direction	x‐direction y‐direction	x‐direction y‐direction				
Median (mm)	0.0	−0.1	0.2	0.5	0.3	0.4	0.5	0.4
Range (mm)	(−3.7, 5.1)	(−3.7, 0.7)	(−1.4, 3.8)	(−1.0, 7.5)	(2.2, 3.4)	(−1.0, 1.2)	(−1.7, 3.7)	(−1.1, 1.1)

Median motion displacements ranged from 0.6 mm in the anterior‐posterior (AP) to 2.9 mm in the left‐right (LR) directions (Table 2). Four of nine DIBH scans showed the largest displacements > 5 mm: 5.2 mm (LR) and 6.3 mm (LR) in P1, 5.9 mm (SI) in P2, and 5.4 mm (SI) in P4. The largest displacement during FB was 5.6 mm (LR) in P5, 7.4 mm (LR) and 6.2 mm (SI) in P6. In two patients subjected to AC (P8, P9), largest displacements stayed below 5 mm, while another patient (P7) reached 10.4 mm (SI) and 6.3 mm (AP). For the patient undergoing HFV (P10), the largest motion was 6.6 mm (LR) (Supplementary Fig. S1).

**Table 2:** Three-dimensional motion displacement across motion management groups (5th–95th percentile). Abbreviation: LR = left‐right; SI = superior‐inferior; AP = anterior‐posterior.

	Deep inspiratory breath‐hold			Free breathing		
	LR	SI	AP	LR	SI	AP
Median (mm)	1.2	1.4	0.6	2.6	1.2	1.4
Range (mm)	(0.1, 6.3)	(0.1, 5.9)	(0, 3.7)	(0.2, 7.4)	(0.1, 6.2)	(0.1, 4.9)

	Abdominal compression			High‐frequency ventilation		
	LR	SI	AP	LR	SI	AP
Median (mm)	1.1	2.5	1.2	2.9	1.2	0.6
Range (mm)	(0.1, 4.6)	(0.1, 10.4)	(0.1, 6.3)	(0.3, 6.6)	(0.1, 3.5)	(0.1, 1.8)

The median (range) fraction of 3D estimations with errors < 2 mm in all axes was 78% (43–96%) using filtered segmentations, compared with 65% (28–89%) using non-filtered segmentations (Wilcoxon signed‐rank test, p < 0.01). Median estimation errors ranged from 0 mm in SI to − 0.5 mm in LR. Large errors occurred in P1 (5.1 mm LR), P6(4.9 mm SI), and P10 (5.0 mm AP) (Table 3).

**Table 3:** Three‐dimensional estimation errors across motion management groups (5th–95th percentile). Abbreviation: LR = left‐right; SI = superior‐inferior; AP = anterior‐posterior.

	Deep inspiratory breath‐hold			Free breathing		
	LR	SI	AP	LR	SI	AP
Median (mm)	0.4	0.0	0.2	−0.5	0.3	−0.1
Range (mm)	(−0.9, 5.1)	(−2.5, 0.4)	(−2.2, 2.6)	(−4.5, 1.4)	(−0.7, 4.9)	(−1.7, 4.0)

	Abdominal compression			High‐frequency ventilation		
	LR	SI	AP	LR	SI	AP
Median (mm)	0.0	0.2	0.1	0.0	0.3	0.1
Range (mm)	(−2.0, 2.6)	(−0.7, 0.8)	(−3.2, 3.8)	(−3.0, 3.7)	(−0.9, 0.8)	(−4.8, 5.0)

## 4 Discussion

This study developed a cardiac and respiratory motion tracking framework using kV images on a gantry‐mounted linear accelerator. This framework, which incorporated a 2D segmentation and a 2D to 3D conversion approach, was implemented retrospectively. ICD lead tip motion was measured under different motion management strategies, revealing patient‐specific variability. These results demonstrate the potential of 3D motion tracking for ICD lead tips during STAR.

The 2D segmentation results demonstrated that the proposed segmentation method is promising. Bertholet et al. reported a 7% false segmentation rate using a template‐based method for arbitrarily shaped markers implanted in the lungs [27]. Our success rate (Table 1) for lead tips was lower than those reported, but this result might be expected as the lead tips were not designed specifically for X‐ray imaging as the case for fiducial markers. Additionally, the Bertholet study was conducted on different datasets with varying imaging conditions. Artifacts such as overlapping leads and wires caused false segmentations in P4 and P6. However, when artifacts differed sufficiently in size or orientation from the lead tip, our method could distinguish the true lead tip, as seen in P2 and P5. This suggests the method is reasonably robust to common ICD carriers and imaging artifacts.

The largest 3D estimation errors occurred in patients with the largest 2D segmentation errors (P1, P6, and P10 Table 1, Table 3), highlighting the importance of precise 2D segmentation. P1 and P10 showed moderate segmentation accuracy, primarily due to low contrast, which contributed to extreme 2D errors as previously discussed [27], [28], and consequently resulted in poor 3D estimations. Exploring deep learning techniques could potentially enhance segmentation performance and further improve 3D tracking accuracy [29].

The Gaussian PDF method was clinically utilized for estimating prostate [30] and liver target motion in real time [31], and mediastinal lymph nodes motion offline in lung cancer patients [32]. In this study, the Gaussian PDF method was applied to estimate lead tip motion with a fast and accurate approach. The clinical application to cardiac and respiratory motion tracking during STAR offers valuable insights and capabilities that have not been demonstrated previously.

During DIBH, motion displacements were slightly smaller than values reported in previous studies: 6.1 (1.2–15.2) mm LR, 3.5 (1.0–9.3) mm SI, and 3.8 (1.4–8.5) mm AP using 4D CT [33] and 3.5 (0–8.1) mm LR, 1.9 (0.1–4.1) mm SI, 1.5 (0–3.9) mm AP using ECG‐gated CT [19]. It should be noted that our measurements represent displacement relative to the mean position. Four of nine DIBH scans showed maximum displacements exceeding 5 mm, whereas one study reported cardiac motions were within 5 mm using cardiac magnetic resonance images [16]. However, differences across scans were observed in our cohort (Supplementary Fig. S1), suggesting variations in lung volume between breath‐holds. Compared with Prusator et al. [12] (3.9 (1.7–6.9) mm LR, 4.7 (2.2–7.9) mm SI, and 4.1 (2.2–5.4) mm AP), abdominal compression motion in this study showed smaller LR and AP motion, but similar SI motion. Since Prusator et al. tracked targets, location‐dependent variability may contribute to the differences, highlighting the need for a correlation model to relate lead tips to target motion. For patients undergoing FB, motion was larger than those observed during AC and DIBH. Knybel et al. [17] reported the largest motion of 6.7 mm (LR), 9.5 mm (SI), and 5.5 mm (AP) during FB, and the present study showed similar values in LR and AP, but slightly lower in SI. Since the data was derived from two patients, it may not fully represent the broader population. Additional scans are needed to validate the findings.

A limitation of this study was the small cohort, reflecting the early‐stage nature of arrhythmia radioablation. Ten patients were analysed, limiting the generalisability of the findings. Future studies with more patients across different breathing patterns are needed to strengthen these findings. However, the patients were recruited under RAVENTA study, this real‐world cohort allowed assessment of the proposed method under clinically relevant conditions.

Projections with poor lead visibility were excluded from the analysis, raising potential concerns about selection bias. However, in clinical practice, if the ICD lead tip is validated as a reliable surrogate for motion tracking, imaging parameters would be optimized to ensure adequate visibility. Therefore, the exclusion reflects a realistic clinical scenario in which image quality would be tailored to support accurate lead‐based tracking.

Another limitation was using the lead tip as a surrogate for target tracking, which requires further investigation. A previous study reported estimates of surrogacy error for tumor tracking in the lung, which may serve as a reference for estimating surrogacy error in STAR trial [18]. Cardiac motion varies across heart regions, and contractile motion may affect radiotherapy precision [33], challenging the assumption that the lead tip consistently reflects target motion. While studies demonstrated its feasibility [12], [21], future research should examine lead tip motion across regions. This may clarify whether lead tips are universally reliable surrogates. To establish a correlation model between lead tips and target motion, multiple types of data will need to be studied. If it can be demonstrated that respiratory and cardiac motion can be decoupled, respiratory motion could be captured using 4D‐CT, and cardiac motion could be modelled using breath‐hold 4D‐CT combined with ECG data. Future work will explore these approaches to quantify and mitigate uncertainties when applying lead tip motion tracking to targets. In this study, 4D‐CT imaging was not available for these patients, as it was not routinely acquired for the participating sites. Future work will compare motion traces with 4D‐CT data once available.

Estimating 3D cardiac motion from time‐resolved 2D projections is challenging due to limited angular sampling, and the use of the lead tips may not fully capture the non‐rigid myocardial deformation. These limitations suggest that the lead tip serves as an indirect motion surrogate rather than a precise representation of the target. Nonetheless, this method remains a viable solution for motion tracking when direct visualization of the ablation target is not feasible. Future studies could incorporate deformable motion models or multiple surrogates.

This framework was implemented retrospectively but shows the potential for real‐time applications. This study focused on the model construction step: the lead tip was segmented from kV images to construct a 3D estimation model prior to treatment. During real-time application, the pre‐trained model could estimate the 3D motion based on segmented 2D positions in continuously acquired kV images during the arc. Future work will investigate the accuracy and feasibility of this approach in real‐time scenarios.

## Author contributions

WX led the data analysis, code and figure generation, writing, and editing of the text. ROB conceived of and led the study, contributed to the conception of ideas, and editing of the text. DTN, DWM, PJK, and CS contributed to the study design, and editing of the text. OB, JBH, and LK contributed to the editing of the text. MG, HJ, SC, MF, MR, OB, JBH, and LK contributed to data generation. WX, DTN, ROB, and LK verified the underlying data.

## Funding

This research was funded by National Health and Medical Research Council (10.13039/501100000925NHMRC) Synergy Grant of Australia (No. 2018592).

## Declaration of competing interest

The authors declare the following financial interests/personal relationships which may be considered as potential competing interests: The authors declare the following financial interests/personal relationships which may be considered as potential competing interests: PJK holds a patent for 2D to 3D estimation (US8379794B2) that has been licensed by Stanford University to Varian Medical Systems. PJK, DTN, and ROB are inventors on additional patents/patent applications related to the KIM technology that have been assigned to the company SeeTreat. PJK and DTN are founders and directors of SeeTreat. OB reports funding from the European Union’s Horizon 2020 STOP‐STORM Consortium project. LK reports on personal fees from AstraZeneca, outside of the submitted work. JBH reports personal fees from EBAMed SA, grants from Elekta AB, personal fees from AstraZeneca, outside of the submitted work. All other authors have no interest to disclose.
