# Supplementary material for: Cardiac device motion tracking from kilovoltage projections during stereotactic arrhythmia radioablation
Source: Phys Imaging Radiat Oncol. 2026 Feb 10;37:100922. doi: 10.1016/j.phro.2026.100922 (PMC12925050; doi:10.1016/j.phro.2026.100922)
Supplement: Supplementary Data 1 [file mmc1.pdf]

Supplementary Table S1

Implantable cardioverter-defibrillator = ICD. P1_1, P1_2, and P1_3 correspond to the first, second, and third scans of patient P1, respectively. Similar labels (e.g., P2_1, P2_2, P3_1, etc.) are used for other patients.

ID	ICD lead	Scan	NO. Projection						
			Total	Lead tips were within the field of view	Percentage (%)	Lead tips were visible by eye	Percentage (%)	Filtered segmentations	Rejection rate (%)
	Deep inspiratory breath-hold								
P1	Medtronic Sprint Quattro Secure S	P1_1	373	267	72	188	50	175	86
		P1_2	714	491	69	351	49	329	94
		P1_3	371	259	70	185	50	178	96
P2	Medtronic Sprint Quattro Secure S	P2_1	368	368	100	358	97	328	92
		P2_2	364	295	81	284	78	269	95
P3	Medtronic Sprint Quattro Secure S	P3_1	184	184	100	140	76	134	96
		P3_2	189	189	100	122	65	105	86
P4	St. Jude Medical Durata	P4_1	368	296	80	295	80	285	97
		P4_2	366	260	71	254	69	247	97
	Free breathing								
P5	Biotronik Plexa ProMRI S DX 65/15	P5_1	352	352	100	332	94	324	98
P6	Boston Scientific, Q-Trek	P6_1	686	686	100	420	61	268	64
	Abdominal compression								
P7	Biotronik Plexa MRI S65	P7_1	661	661	100	447	68	400	89
P8	Boston Scientific Reliance G Dual Coil	P8_1	326	326	100	326	100	297	91
P9	Medtronic, Sprint Quattro Secure S	P9_1	376	376	100	303	81	242	80
	High frequency ventilation								
P10	Boston Scientific, RELIANCE S Single-Coil	P10_1	680	558	82	419	62	333	79
	Median		368	327	100	303	70	269	93
	Min		184	184	69	122	49	105	64
	Max		714	686	100	447	100	400	98
	Total		6378	5568		4424		3914	

Supplementary Figure S1

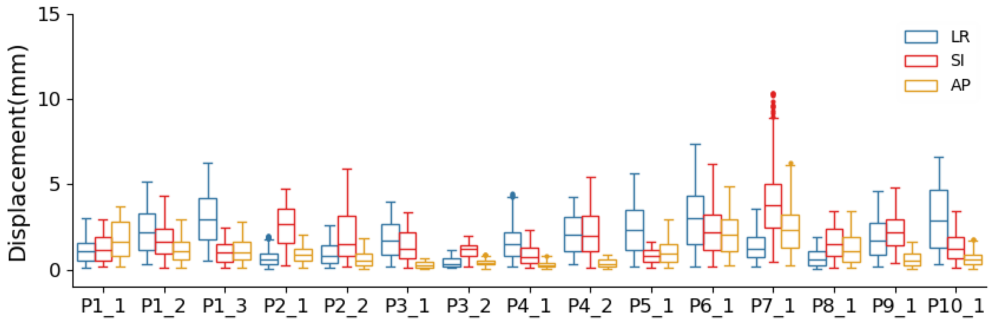

Figure 1: Implantable cardioverter-defibrillator lead tip motion displacement. Abbreviation: LR = left-right; SI = superior-inferior; AP = anterior-posterior.
